# Fluoroquinolones Are Useful as Directed Treatment for Complicated UTI in a Setting with a High Prevalence of Quinolone-Resistant Microorganisms

**DOI:** 10.3390/antibiotics12010183

**Published:** 2023-01-16

**Authors:** Arturo Artero, Ian López-Cruz, Laura Piles, Juan Alberola, José María Eiros, Sofia Salavert, Manuel Madrazo

**Affiliations:** 1 Hospital Universitario Doctor Peset, 46017 Valencia, Spain; 2 Hospital Universitario Rio Hortega, 47012 Valladolid, Spain

**Keywords:** community-acquired UTI, risk factors, fluoroquinolones, resistance, outcomes

## Abstract

Fluoroquinolones (FQs) have been widely used for treating urinary tract infections (UTIs); however, the increasing emergence of resistant strains has compromised their use. We aimed to know the usefulness of FQs for the treatment of community-acquired UTI in a setting with a high prevalence of fluoroquinolone-resistant microorganisms. A prospective observational study of patients diagnosed with community-acquired UTI was conducted, in which their outcomes according to whether they had FQs or not in their empirical and directed treatments were compared. A multivariate analysis was performed to identify risk factors for UTIs due to ciprofloxacin-resistant microorganisms. A total of 419 patients were included; 162 (38.7%) patients were treated with FQs, as empirical treatment in 27 (6.4%), and as directed treatment in 135 (32.2%). In-hospital mortality (2.2% vs. 6.6%, *p* 0.044) and 30-day mortality (4.4 vs. 11%, *p* 0.028) were both lower in the group of patients directly treated with FQ, while there were no differences when FQs were used as empirical treatment. A total of 37.2% of the cases were resistant to ciprofloxacin, which was associated with healthcare-associated UTI (OR 2.7, 95% CI 2–3.7) and prior exposure to FQs (OR 2.7, 95 % CI 1.9–3.7). In conclusion, our findings show that in a setting with a high prevalence of community-acquired UTI caused by quinolone-resistant microorganisms, FQs as directed treatment for community-acquired UTI were associated with better outcomes than other antibiotics, but their use as empirical treatment is not indicated, even in those cases without risk factors for quinolones resistance.

## 1. Introduction

Urinary tract infections (UTIs) are amongst the most frequent bacterial infections in the community with considerable morbidity [1], and require antibiotic treatment. Fluoroquinolones (FQs) have been extensively used as empirical treatment for UTI [2]; that is, before the confirmation of the etiology and antibiogram, since their spectrum of activity includes enteric Gram-negative bacilli [3]. They are recommended as directed treatment [2] once the bacteria that cause the infections and their antibiogram are known, since they have many beneficial pharmacokinetic properties [3]. In addition, FQs may be used as shorter antibiotic courses for complicated UTIs [2,4,5].

However, their use has been compromised by the increasing emergence of resistant strains [6,7,8]. Resistance to fluoroquinolones is multifactorial and can be via one or a combination of target-site gene mutations, increased production of efflux pumps, modifying enzymes, and/or target-protection proteins [9]. The most common mechanism of resistance is the mutation in one or more of the genes that encode type II topoisomerases (which include DNA gyrase and topoisomerase IV) and alter the fluoroquinolones binding affinity of the enzyme [10]. Although fluoroquinolones preferentially target either DNA gyrase or topoisomerase IV for Gram-negative or Gram-positive bacteria, respectively, they will bind to the secondary target, which in turn becomes a target once the primary binding is mutated [11]. Physicians should be aware of risk factors associated with FQ resistance, the most important of which is prior exposure to FQs [7,12,13].

In addition, although FQs are usually well tolerated [14], they can cause uncommon but potentially permanent and disabling adverse effects involving the musculoskeletal and nervous systems, leading to restrictions in their use for uncomplicated infections [15] and being reserved for severe infections, such as complicated UTIs.

Despite their beneficial characteristics, increasing resistance and potential adverse effects may lead to the phasing out of the use of FQs. In this work, we aimed to know the usefulness of FQs for both the empirical and directed treatment of complicated community-acquired UTI in a setting with a high prevalence of quinolone-resistant microorganisms, and to know whether they are more suitable in patients with certain epidemiological and clinical characteristics.

## 2. Material and Methods

### 2.1. Patient Selection

Cohort prospective observational study of patients consecutively admitted to an internal medicine ward at a university hospital, diagnosed with community-acquired UTI, from January 2017 to December 2021. Nosocomial or UTI cases transferred from the intensive care unit (ICU), as well as cases with a negative urine culture or a clinical syndrome compatible with any other condition, after being reviewed by two independent researchers, were excluded. Epidemiological and clinical variables were collected by the authors following a protocol [16]. This study was approved by the Clinical Research Ethics Committee of the Doctor Peset University Hospital (code 85/16, September 2016) and followed the STROBE statement.

The microbial identifications of the urine cultures were made using the Bruker MALDI Biotyper system (Beckman Coulter, Brea, CA, USA), and for the drug sensitivity and resistance tests, the DxM MicroScan WalkAway microbiology system (Beckman Coulter, Brea, CA, USA) was used. This is a microbroth dilution method based on a combination of CLSI and EUCAST rules. Inadequate empiric antimicrobial therapy (IEAT) was considered as the occurrence of infection that was not effectively treated at the time when the causative microorganism and its antimicrobial susceptibility were known. This included the absence of antimicrobial agents directed at a specific class of microorganisms and the administration of an antimicrobial agent to which the microorganism responsible for the infection was resistant [17].

Community-onset healthcare-associated UTI (HCA-UTI) was defined as a community-onset UTI with any of the following criteria: (I) having been admitted to an acute care hospital ≥48 h within the 90 days prior to current hospital admission; (II) having received antimicrobial therapy within the 90 days prior to current hospital admission; and (III) residing in a nursing home [18]. Community-acquired infection was defined when the symptoms of the urinary infection were initiated in the community and none of the previous criteria were met [18]. UTI was considered as complicated when the patient had either structural or functional urinary tract abnormalities, with a high likelihood of treatment failure and serious complications [19].

### 2.2. Data Analysis

Quantitative variables were compared using Student’s t-test or analysis of variance (ANOVA) when the distribution was normal, or Mann–Whitney U-test when it was not normal. Qualitative variables were compared with the chi-square test and Fisher’s exact test. Multivariate analysis was performed using logistic regression, considering an α significance level of 0.05 for all tests. The statistical package SPSS version 22 from IBM for Windows was used for the statistical analysis.

## 3. Results

**Figure 1:**
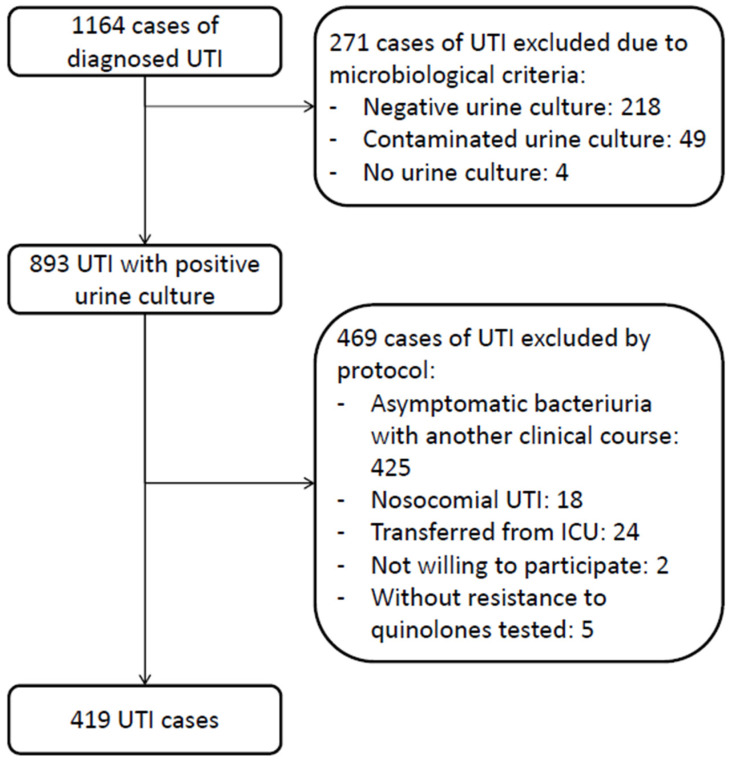
Flowchart of inclusion of 419 cases of complicated community-acquired urinary tract infection.

Of the total 1164 patients with community-acquired UTI admitted to the hospital, 419 were included in this study (Figure 1). A total of 162 (38.7%) patients were treated with FQs; 27 (6.4%) were given FQs as empirical treatment (20 patients were treated with levofloxacin and 7 with ciprofloxacin), and 135 (32.2%) as directed treatment (21 patients with levofloxacin and 114 with ciprofloxacin). The median age of the patients was 78 (70–86) years, and 51.6% were female. Diabetes mellitus (36%), moderate chronic kidney disease (32.5%), and dementia (26.7%) were the most common comorbidities, and 88.1% of the patients had a Charlson comorbidity index of 3 or more. There were no significant differences between the empirical treatment with FQs (ETQ) group and the empirical treatment with other antibiotics (ETOA) group regarding epidemiological and clinical variables (see Table 1). The patients in the directed treatment with FQs (DTQ) group had fewer HCA-UTIs than those in the directed treatment with other antibiotics (DTOA) group (DTQ 41.2% vs. DTOA 59.9%, *p* < 0.001) and fewer cases caused by multi-drug resistant (MDR) bacteria (DTQ 25% vs. DTOA 30.7%, *p* 0.005).

The most common antibiotic therapy in the ETOA group was ceftriaxone (48.7%), followed by meropenem (17.9%), gentamicin plus a beta-lactam antibiotic (9.2%), amoxicillin/clavulanic acid (5.9%), ertapenem (3.3%), piperacillin/tazobactam (1.8%), aztreonam (1.8%), and fosfomycin (1%). Ceftriaxone was also the most common antibiotic therapy in the DTOA group (21.6%), followed by cefuroxime (19%), meropenem (15.4%), amoxicillin/clavulanic acid (10.6%), ertapenem (10.6%), piperacillin/tazobactam (3.7%), fosfomycin (3.7%), gentamicin plus a beta-lactam antibiotic (1.5%), and aztreonam (1.1%).

A total of 458 microorganisms were isolated in the urine cultures; *Escherichia coli* was the most common microorganism (64.7%), followed by *Klebsiella pneumoniae* (12.9%), *Enterococcus faecalis* (8.1%), *Pseudomonas aeruginosa* (6.4%), and *Proteus mirabilis* (4.1%). A total of 8.6% of the patients were found to be infected with two or more types of bacterial isolates at once.

Resistance to ciprofloxacin was found in 156 cases (37.2%). The epidemiological, clinical and microbiological variables associated with resistance to ciprofloxacin by univariate analysis are shown In Table 2. Prior exposure to FQs (OR 2.7, 95% CI 1.9–3.7) and HCA-UTI (OR 2.7, 95% CI 2–3.7) were independently associated with resistance to ciprofloxacin by multivariate analysis (see Table 3). While 37.2% of the cases were resistant to ciprofloxacin in the total of the series, the percentage of resistance in the cases with prior exposure to FQs or HCA-UTI was 52.4%, as compared to 19.3% in those cases without any of these two risk factors (*p* < 0.001).

In-hospital mortality was 7.6%, and 30-day mortality was 11.2%, with no difference between the ETQ and the ETOA groups. Eleven patients died before receiving directed antibiotic treatment. Mortality was significantly lower in the DTQ group in both in-hospital mortality (DTQ 2.2% vs. DTOA 6.6%, *p* 0.044) and 30-day mortality (DTQ 4.4% vs. DTOA 11%, *p* 0.028). However, the length of hospital stay was not different between patients treated with FQs and those with other antibiotics (DTQ 5 (3–7) days vs. DTOA 5 (3–7) days, *p* 0.328).

## 4. Discussion

In this observational study conducted in a high prevalence of quinolone-resistant microorganisms setting, the use of FQs as directed treatment for complicated community-acquired UTIs resulted in better outcomes than other antibiotics. As was expected, the use of FQs as empirical treatment was scarce, but did not result in a worse prognosis.

In the present study, resistance to ciprofloxacin among microorganisms causing complicated community-acquired UTIs was 37.2%. The findings show a higher rate of quinolone resistance than those found in other studies conducted in Europe and the United States, which include community-acquired uncomplicated lower UTI, complicated lower UTI, and pyelonephritis, ranging between 23 and 31% [8,20,21,22,23]. The high percentage of quinolone-resistant UTIs observed in this study could be explained by the high prevalence of community-acquired HCA-UTIs (54.4%) in a population with many comorbidities.

As recommended by international guidelines, an FQ is not an appropriate choice for empiric therapy in patients with complicated community-acquired UTI where the prevalence of FQ resistance is thought to exceed 10% [2]. According to that, they were used in only 6.4% of the patients in our study. This rate is at variance with previous studies published after the release of the 2016 FDA warning [15], in which the rates of fluoroquinolone prescribed for complicated and uncomplicated UTIs varied from 21 to 42% [24,25]. 

In the present study, there were no differences in IEAT between ETQ and ETOA groups (29.6% vs. 21.9%, *p* 0.350), nor were there any in outcomes. The lack of influence on the prognosis of empirical treatment with FQs compared to other empirical antibiotics may be due to the small number of patients who received FQs and to the high use of beta-lactams that result in a high percentage of IEAT (21.9%).

Identifying the risk factors that predispose individuals to infection by microorganisms resistant to quinolones is of great importance in a setting with a high prevalence of quinolone-resistant microorganisms. Prior exposure to quinolones is the main risk factor for quinolone resistance, as shown in our study and others [7,12,13,26]. Interestingly, the results show that HCA-UTI was also independently associated with quinolone resistance in this study. The three factors defining HCA-UTI were associated with resistance to ciprofloxacin in the present study, as has been shown in a recent systematic review [26]. Thus, previous use of antibiotics, residence in a nursing home, and previous hospitalization should be considered for the choice of empirical antimicrobial therapy. Other risk factors for quinolone resistance, such as an indwelling urinary catheter, sex, and older age, have been pointed to in some studies [7,13,23], but they showed no independent association with quinolone resistance in our study, as in others [12,26].

When patients with or without a history of prior use of quinolones or an HCA-UTI regarding resistance to quinolones were compared, the results (52.4% vs. 19.3%, respectively, *p* < 0.001) were statistically significant, but 19.3% is still above the 10% recommended to use quinolones as empirical treatment [2,27]. Clinical decisions on the choice of FQs as initial empirical therapy would be best informed initially by local susceptibility data and later by specific susceptibility data on the strain isolated in each case. Resistance to quinolones shares risk factors with resistance to other antibiotics [23]. In the present study, resistance to quinolones was related to MDR and extended-spectrum beta-lactamase (ESBL) producing microorganisms (65.4% and 23.1% of the patients with UTI due to ciprofloxacin-resistant bacteria, respectively). These findings are consistent with the results of previous studies that found an association of quinolone resistance with MDR [28] and ESBL-producing microorganisms in UTIs [29]. Therefore, considering empirical treatment with carbapenems for complicated UTIs in patients with risk factors for MDR pathogens should be noted [29].

The efficacy of quinolones has been widely established in complicated UTIs as directed therapy. In the present study, 32.1% of the patients had quinolones as directed antibiotic therapy. Interestingly, FQs were associated with lower mortality in both in-hospital and 30-day mortality, reflecting the efficacy of quinolones seen in other studies [4,7,14,30]. However, since patients who received FQs had fewer comorbidities, less HCA-UTI, lower IEAT, and a slightly better functional status, we cannot rule out that these factors had influenced mortality. Patients in the DTQ group had the same length of hospital stay as those in the DTOA group. However, whether the DTQ group had a shorter duration of treatment as is presumed and recommended when using FQs as directed therapy could not be analyzed [2,27].

The present study has certain limitations. First, conducting this study at a single center limits generalizability. Second, this was an observational study, and therefore the findings do not prove causality. Additionally, third, the antecedent of recurrent UTI was not included in this study; it has been pointed out as a risk factor for ciprofloxacin resistance in some studies [7,12] but not in others [23]. However, we believe that our findings add knowledge to the usefulness of quinolones as empirical and directed treatment for complicated community-acquired UTIs, which can lead to better management of community-acquired complicated UTIs.

## 5. Conclusions

The results of this observational study indicate that in a setting with a high prevalence of quinolone-resistant microorganisms, the use of FQs as directed treatment for complicated community-acquired UTI was associated with better outcomes than other antibiotics. However, their use as empirical treatment is not indicated, even in patients without risk factors for quinolone resistance, although it did not result in a worse prognosis.

## Figures and Tables

**Table 1:** Epidemiological and clinical characteristics and outcomes of community-acquired urinary tract infection according to use of fluoroquinolones as empiric or directed antibiotic therapy.

	Total *n* 419	ETQ *n* 27	ETOA *n* 388 *	*p*	DTQ *n* 135	DTOA *n* 273 **	*p*
Female, *n* (%)	216 (51.6)	14 (51.9)	198 (51)	0.934	67 (49.6)	142 (52)	0.650
Age (years), median (IQR)	79 (70–86)	79 (71–85)	79 (70–87)	0.935	77 (67–86)	79 (71–87)	0.136
Age ≥ 75 years, *n* (%)	269 (64.2)	18 (66.7)	249 (64.2)	0.794	77 (57)	183 (67)	0.048
McCabe ≥ 2, *n* (%)	294 (70.2)	20 (74.1)	271 (69.8)	0.643	76 (56.3)	207 (75.8)	<0.001
Charlson ≥ 3, *n* (%)	369 (88.1)	23 (85.2)	343 (88.4)	0.616	115 (85.2)	243 (89)	0.267
Barthel < 40, *n* (%)	137 (32.7)	10 (37)	127 (32.7)	0.646	29 (21.5)	99 (36.3)	0.002
Comorbidities							
Dementia, *n* (%)	112 (26.7)	8 (29.6)	104 (26.8)	0.749	28 (20.7)	76 (27.8)	0.122
Diabetes mellitus, *n* (%)	151 (36)	13 (48.1)	136 (35.1)	0.170	46 (34.1)	100 (36.6)	0.612
COPD, *n* (%)	51 (12.2)	3 (11.1)	48 (12.4)	0.847	15 (11.1)	35 (12.8)	0.620
CKD, *n* (%)	136 (32.5)	10 (37)	125 (32.2)	0.605	37 (27.4)	95 (34.8)	0.133
Cancer, *n* (%)	88 (21)	3 (11.1)	83 (21.4)	0.203	30 (22.2)	55 (20.1)	0.627
Indwelling urinary catheter, *n* (%)	81 (19.3)	3 (11.1)	77 (19.8)	0.265	22 (16.3)	58 (21.2)	0.236
HCA-UTI, *n* (%)	227 (54.2)	17 (63)	208 (53.6)	0.345	56 (41.5)	163 (59.7)	0.001
Previous hospitalization, *n* (%)	127 (30.3)	7 (25.9)	119 (31)	0.604	28 (20.7)	93 (34.1)	0.006
Previous antimicrobial therapy, *n* (%)	192 (45.8)	16 (59.3)	174 (44.8)	0.146	53 (39.3)	133 (48.7)	0.044
Nursing home residence, *n* (%)	27 (6.4)	0	27 (7)	0.156	2 (1.5)	25 (9.2)	0.003
Prior exposure to quinolones, *n* (%)	47 (11.2)	3 (11.1)	44 (11.3)	0.971	8 (5.9)	39 (14.3)	0.016
Clinical characteristics							
APACHE II, median (IQR)	11 (8–16)	10 (8–12)	11 (8–16)	0.167	10 (8–14)	11 (8–16)	0.146
Fever, *n* (%)	329 (78.5)	22 (81.5)	304 (78.4)	0.702	113 (83.7)	207 (75.8)	0.069
RR ≥ 22, *n* (%)	79 (18.9)	3 (11.1)	74 (19.1)	0.301	24 (17.8)	47 (17.3)	0.901
Altered mental status, *n* (%)	169 (40.3)	8 (29.6)	159 (41.1)	0.241	38 (28.1)	122 (44.9)	0.001
SBP < 100, *n* (%)	75 (17.9)	3 (11.1)	72 (18.6)	0.328	24 (17.8)	46 (16.9)	0.827
qSOFA ≥ 2, *n* (%)	101 (24.1)	5 (18.5)	95 (24.5)	0.483	23 (17)	68 (24.9)	0.072
Sepsis (SOFA ≥ 2), *n* (%)	175 (41.8)	7 (25.9)	166 (42.8)	0.086	52 (38.5)	113 (41.4)	0.578
Septic shock-3, *n* (%)	38 (9.1)	0	38 (9.8)	0.088	9 (6.7)	22 (8.1)	0.618
Albumin, median (IQR)	3.3 (3–3.6)	3.4 (3.1–3.5)	3.3 (3–3.6)	0.645	3.4 (3.1–3.6)	3.3 (2.9–3.6)	0.110
Leukocytosis, median (IQR)	13,100 (9300–18,100)	13,200 (9400–14,100)	13,100 (9300–18,400)	0.646	13,500 (9400–17,900)	12,700 (8900–18,000)	0.363
Polymicrobial UTI, *n* (%)	36 (8.6)	1 (3.7)	35 (9)	0.343	8 (5.9)	26 (9.5)	0.216
MDR, *n* (%)	148 (35.3)	13 (48.1)	135 (34.8)	0.161	34 (25.2)	110 (40.3)	0.003
BLEE, *n* (%)	40 (9.5)	4 (14.8)	36 (9.3)	0.346	3 (2.2)	37 (13.6)	<0.001
Bacteremia, positive/total blood cultures, *n* (%)	90/236 (38.1)	5/13 (38.5)	84/221 (38)	0.529	28/81 (34.6)	58/148 (39.2)	0.533
IEAT, *n* (%)	92 (22)	8 (29.6)	82 (21.1)	0.300	13 (9.6)	76 (27.8)	<0.001
Outcomes							
In-hospital mortality, *n* (%)	32 (7.6)	3 (11.1)	29 (7.5)	0.493	3 (2.2)	18 (6.6)	0.044
30-day mortality, *n* (%)	47 (11.2)	5 (18.5)	42 (10.8)	0.223	6 (4.4)	30 (11)	0.028
Length of hospital stay (days), median (IQR)	5 (3–7)	5 (3–6)	5 (3–7)	0.571	5 (3–7)	5 (3–7)	0.328

ETQ, empirical treatment with fluoroquinolones; ETOA, empirical treatment with other antibiotics; DTQ directed treatment with other antibiotics; DTOA, directed treatment with other antibiotics; MDR, multidrug-resistant; COPD, chronic obstructive pulmonary disease; CKD, chronic kidney disease; HCA-UTI, healthcare associated-UTI; RR, respiratory rate; SBP, systolic blood pressure; IEAT, inadequate empiric antimicrobial therapy; * 4 patients received directed therapy from baseline based on recent culture results; ** 11 patients died before changing their treatment to directed antibiotic therapy.

**Table 2:** Risk factors for resistance to ciprofloxacin of complicated community-acquired urinary tract infection.

	Resistance to Ciprofloxacin *n* 156	Non-resistance to Ciprofloxacin *n* 263	*p*
Female, *n* (%)	71 (45.5)	145 (55.1)	0.057
Age (years), median (IQR)	81 (74–87]	77 (66–85)	0.002
Age ≥ 75 years, *n* (%)	115 (73.7)	154 (58.6)	0.002
McCabe ≥ 2, *n* (%)	125 (80.1)	169 (64.3)	0.001
Charlson ≥ 3, *n* (%)	146 (93.6)	223 (84.8)	0.007
Barthel < 40, *n* (%)	68 (43.6)	69 (26.2)	<0.001
Comorbidities			
Dementia, *n* (%)	48 (30.8)	64 (24.3)	0.150
Diabetes mellitus, *n* (%)	62 (39.7)	89 (33.8)	0.224
COPD, *n* (%)	24 (15.4)	27 (10.3)	0.121
CKD, *n* (%)	66 (42.3)	70 (26.6)	0.001
Cancer, *n* (%)	35 (22.4)	53 (20.2)	0.579
Indwelling urinary catheter, *n* (%)	46 (29.5)	35 (13.3)	<0.001
Prior exposure to quinolones, *n* (%)	35 (22.4)	12 (4.6)	<0.001
HCA-UTI, *n* (%) Previous hospitalization, *n* (%) Previous antimicrobial therapy, *n* (%) Nursing home residence, *n* (%)	119 (76.3) 68 (43.6) 102 (65.4) 21 (13.5)	108 (41.1) 59 (22.4) 90 (34.2) 6 (2.3)	<0.001 <0.001 <0.001 <0.001
Severity scores			
APACHE II, median (IQR)	12 (8–17)	11 (8–14)	0.016
qSOFA ≥ 2, *n* (%)	45 (28.8)	56 (21.3)	0.081
Sepsis (SOFA ≥ 2), *n* (%)	65 (41.7)	110 (41.8)	0.975
Septic shock-3, *n* (%)	17 (10.9)	21 (8)	0.316
Microbiological data			
Polymicrobial UTI, *n* (%)	19 (12.2)	17 (6.5)	0.044
MDR, *n* (%)	102 (65.4)	46 (17.5)	<0.001
ESBL, *n* (%)	36 (23.1)	4 (1.5)	<0.001
Bacteremia, positive/total blood cultures, *n* (%)	35/86 (40.7)	55/150 (36.7)	0.891

COPD, chronic obstructive pulmonary disease; CKD, chronic kidney disease; HCA-UTI, healthcare-associated urinary tract infection; MDR, multidrug-resistant bacteria; ESBL, extended-spectrum beta-lactamase-producing *Enterobacteriaceae*; IEAT, inadequate empiric antibiotic therapy.

**Table 3:** Multivariate analysis of risk factors for resistance to ciprofloxacin of complicated community-acquired urinary tract infection.

	Univariate Analysis *p*	OR (95% CI)	Multivariate Analysis *p*	OR (95% CI)
Charlson ≥ 3	0.007	1.9 (1.1–3.5)	0.866	-
Age ≥ 75 years old	0.002	1.6 (1.2–2.1)	0.157	
Barthel < 40	<0.001	1.6 (1.3–2)	0.064	-
HCA-UTI	<0.001	2.7 (2–3.7)	<0.001	22 (12.4–31.7)
Previous use of quinolones	<0.001	2.7 (1.9–3.7)	<0.001	28.5 (14.2–42.9)
Indwelling catheter	<0.001	1.7 (1.4–2.2)	0.091	-
APACHE II ≥ 12	0.781	1.1 (0.6–1.8)	0.807	-

HCA-UTI, healthcare-associated urinary tract infection.

## Data Availability

The data presented in this study are available on request from the corresponding author. The data are not publicly available due to privacy.
